# Supplementary material for: Signatures of correlation of spacetime fluctuations in laser interferometers
Source: Nat Commun. 2025 Dec 23;17:701. doi: 10.1038/s41467-025-67313-3 (PMC12820127; doi:10.1038/s41467-025-67313-3)
Supplement: Supplementary file 1 — Supplementary Information [file 41467_2025_67313_MOESM1_ESM.pdf]

Supplementary Information for "Signatures of Correlation of Spacetime Fluctuations in Laser Interferometers"

B. Sharmila,[1,*] Sander M. Vermeulen,[2,†] and Animesh Datta[1,‡]

[1] *Department of Physics, University of Warwick, Coventry CV4 7AL, UK.*
[2] *Division of Physics, Mathematics and Astronomy, California Institute of Technology, Pasadena, CA 91125, USA.*

(Dated: November 24, 2025)

I. I. PROPAGATION OF LIGHT IN A FLUCTUATING SPACETIME

In this section, we will solve the relativistic wave equation, applying necessary approximations, to obtain the electric field of light propagating in a fluctuating spacetime.

The relativistic wave equation in terms of the electromagnetic field tensor $F_{\alpha\beta}$ [1]

$$\Box F_{\alpha\beta} + 2R_{\alpha\gamma\beta\delta}F^{\gamma\delta} - R_{\alpha\gamma}F^{\gamma}_{\beta} + R_{\beta\gamma}F^{\gamma}_{\alpha} = 0,$$

where

$$\begin{aligned}\Box F_{\alpha\beta} &= g^{\gamma\delta}\nabla_\gamma\nabla_\delta F_{\alpha\beta}\\ &= g^{\gamma\delta}\Big(\partial_\delta X_{\alpha\beta\gamma} - \Gamma^{\eta}_{\alpha\delta}X_{\eta\beta\gamma} - \Gamma^{\eta}_{\beta\delta}X_{\alpha\eta\gamma}\\ &\qquad - \Gamma^{\eta}_{\gamma\delta}X_{\alpha\beta\eta}\Big),\end{aligned} \tag{1}$$

with

$$X_{\alpha\beta\gamma} = \partial_\gamma F_{\alpha\beta} - \Gamma^{\eta}_{\beta\gamma}F_{\alpha\eta} - \Gamma^{\eta}_{\alpha\gamma}F_{\eta\beta}, \tag{2}$$

$$R_{\alpha\beta\gamma\delta} = g_{\alpha\nu}\left[\partial_\gamma\Gamma^{\nu}_{\delta\beta} - \partial_\delta\Gamma^{\nu}_{\gamma\beta} + \Gamma^{\nu}_{\gamma\eta}\Gamma^{\eta}_{\delta\beta} - \Gamma^{\nu}_{\delta\eta}\Gamma^{\eta}_{\gamma\beta}\right], \tag{3}$$

and

$$R_{\alpha\beta} = R^{\nu}_{\alpha\nu\beta}, \tag{4}$$

with the Christoffel symbol

$$\Gamma^{\alpha}_{\beta\gamma} = g^{\alpha\mu}(\partial_\beta g_{\mu\gamma} + \partial_\gamma g_{\beta\mu} - \partial_\mu g_{\beta\gamma})/2. \tag{5}$$

Here the Greek indices take values from the set $\{0,1,2,3\}$, with 0 corresponding to the timelike component and the rest to spacelike components.

Assuming the most general $g_{\beta\gamma}$ without any further assumptions, it can be trivially seen that it is not possible to simplify the above relativistic equation. Therefore, we consider the eikonal approximation to find a solution to $-cF_{0j}$ $(j = 1,2,3)$ or equivalently, $\vec{E}$, the 3-vector electric field.

* Sharmila.Balamurugan@warwick.ac.uk
† smv@caltech.edu
‡ Animesh.Datta@warwick.ac.uk

(i) *Ansatz:* Let us consider electric field of the form,

$$\vec{E}(\boldsymbol{r}) = \vec{E}_0(\boldsymbol{r})e^{ik\Phi(\boldsymbol{r})}, \tag{6}$$

where $k = 2\pi/\lambda = \Omega/c$ with the wavelength λ and the frequency Ω of the electromagnetic (EM) radiation propagating in the fluctuating spacetime. Also $\boldsymbol{r} \equiv (t,x,y,z)$. To apply the eikonal approximation, we use Eq. (6) in Eq. (1) and consider $k \to \infty$. Note that this sets the wavelength and the time-period of the EM radiation to be the smallest length and time scales respectively in the system.

Using Assumption (i), we find that Eq. (1) reduces to

$$g^{\gamma\delta}\partial_\gamma\partial_\delta F_{\alpha\beta} = 0. \tag{7}$$

This is because in the presence of terms that are 2-order derivatives of $F_{\alpha\beta}$, terms proportional to smaller order derivatives don't survive.

Let us now consider the following form of the metric to simplify this further.

(1) We consider a spacetime metric of the form,

$$g^{\mu\nu} = \eta^{\mu\nu} + 2w^{\mu\nu}, \tag{8}$$

where the 4×4 matrix w is a real, symmetric, matrix that models fluctuations about the flat Minkowski metric $\eta_{\mu\nu}$ with a signature $(-1,1,1,1)$.

Applying both Assumption (i) and Attribute (1), we find

$$\begin{aligned}&-\frac{(1-2w^{00})}{c^2}(\partial_t\Phi)^2 + (1+2w^{11})(\partial_x\Phi)^2\\ &+(1+2w^{22})(\partial_y\Phi)^2 + (1+2w^{33})(\partial_z\Phi)^2\\ &+\frac{4}{c}(\partial_t\Phi)\left[w^{01}(\partial_x\Phi) + w^{02}(\partial_y\Phi) + w^{03}(\partial_z\Phi)\right]\\ &+4(\partial_x\Phi)\left[w^{12}(\partial_y\Phi) + w^{13}(\partial_z\Phi)\right] + 4w^{23}(\partial_y\Phi)(\partial_z\Phi) = 0.\end{aligned} \tag{9}$$

(ii) *Slowly varying envelope approximation (SVEA):* To be consistent with Assumption (i), we also consider the following ansatz,

$$\Phi(\boldsymbol{r}) = ct - z + \Phi_{\text{F}}(\boldsymbol{r}), \tag{10}$$

with $\partial_\mu\Phi_{\text{F}} \ll 1$ $(\mu = 0,1,2,3)$.

Using Assumption (ii), we neglect terms of order $(\partial_\mu \Phi_\mathrm{F})^2$. With this, we find

$$
\begin{aligned}
&-(1-2w^{00}) - \frac{2(1-2w^{00})}{c}(\partial_t \Phi_\mathrm{F}) \\
&+(1+2w^{33}) - 2(1+2w^{33})(\partial_z \Phi_\mathrm{F}) + 4w^{01}(\partial_x \Phi_\mathrm{F}) \\
&+4w^{02}(\partial_y \Phi_\mathrm{F}) + 4w^{03}(\partial_z \Phi_\mathrm{F}) - \frac{4}{c} w^{03}(\partial_t \Phi_\mathrm{F}) - 4w^{03} \\
&-4w^{13}(\partial_x \Phi_\mathrm{F}) - 4w^{23}(\partial_y \Phi_\mathrm{F}) = 0. \qquad (11)
\end{aligned}
$$

(2) We also consider the metric fluctuations to be small, i.e., $w^{\mu\nu} \ll 1$ $(\mu, \nu = 0, 1, 2, 3)$.

Using Assumption (ii) and Attribute (2) together, we neglect terms of order $w^{\mu\nu}(\partial_\gamma \Phi_\mathrm{F})$. With this, we find

$$
-\frac{2}{c}(\partial_t \Phi_\mathrm{F}) - 2(\partial_z \Phi_\mathrm{F}) + 2w^{00} + 2w^{33} - 4w^{03} = 0. \qquad (12)
$$

A general solution is

$$
\begin{aligned}
\Phi_\mathrm{F}(\boldsymbol{r}) = \mathcal{F}(ct - z) + c \int_0^t \mathrm{d}t' \, \big[w^{00}(\boldsymbol{r}(t')) \\
+ w^{33}(\boldsymbol{r}(t')) - 2w^{03}(\boldsymbol{r}(t')) \big], \qquad (13)
\end{aligned}
$$

where $\mathcal{F}$ denotes any general function of the given argument. We choose the following solution as one that best fits our initial conditions of an input Gaussian beam.

$$
\vec{E}(\boldsymbol{r}) = \vec{E}_{in}(x, y) e^{ik\Phi(\boldsymbol{r})}, \qquad (14)
$$

where

$$
\begin{aligned}
\vec{E}_{in}(x, y) = \sqrt{\frac{2}{\pi}} \frac{z_R}{W_0 \sqrt{z_R^2 + z_0^2}} \\
\exp\left[\left(-\frac{ikz_0 W_0^2 + 2z_R^2}{2W_0^2 (z_0^2 + z_R^2)} \right)(x^2 + y^2) \right] \hat{e}_y, \qquad (15a)
\end{aligned}
$$

$$
\begin{aligned}
\Phi(\boldsymbol{r}) = ct - z + c \int_0^t \mathrm{d}t' \, \big[w^{00}(\boldsymbol{r}(t')) + w^{33}(\boldsymbol{r}(t')) \\
-2w^{03}(\boldsymbol{r}(t')) \big], \qquad (15b)
\end{aligned}
$$

with $\hat{e}_y$ being the unit vector along the y-axis, W_0 the beam waist, z_0 the position of the beam waist, and $z_R = \pi W_0^2/\lambda$.

II. II. HOLOMETER-TYPE SETUP: SPECTRAL DENSITIES

In this section, we obtain the power and cross spectral densities of the optical path difference between the two arms of the Michelson laser interferometer (MLI) with no arm cavities.

The electric field at the output port (see Fig. 1) of the Interferometer p ($p = \text{I,II}$) is

$$
\begin{aligned}
E^p_\mathrm{out}(\boldsymbol{r}_\mathrm{D}(\Delta_\tau, \Delta)) = \frac{1}{\sqrt{2}} \Big[E_y^{(\mathrm{C}_p)}(\boldsymbol{r}_\mathrm{D}(\Delta_\tau, \Delta)) \\
- E_y^{(\mathrm{D}_p)}(\boldsymbol{r}_\mathrm{D}(\Delta_\tau, \Delta)) e^{-2i\varphi_\mathrm{off}} \Big]. \qquad (16)
\end{aligned}
$$

Here the detector is at $\boldsymbol{r}_\mathrm{D}(\Delta_\tau, \Delta) \equiv (\tau_0 + \Delta_\tau, \Delta, \Delta, \Delta)$ with $\Delta = 0$ for Interferometer I and $\Delta = \Delta_s$ for Interferometer II. We note that $\tau_0 = 2\mathcal{L}/c$.

As a first step towards finding the spectral densities of the interferometric output, we define electric field correlation tensors of the form,

$$
M^{m,m'}_{\boldsymbol{c};\boldsymbol{c'}}(\{\boldsymbol{r}\};\{\boldsymbol{r'}\}) = E_y^{(c_1)}(\boldsymbol{r}_1) E_y^{(c_2)}(\boldsymbol{r}_2) \cdots E_y^{(c_m)}(\boldsymbol{r}_m) E_y^{(c'_1)*}(\boldsymbol{r'}_1) E_y^{(c'_2)*}(\boldsymbol{r'}_2) \cdots E_y^{(c'_{m'})*}(\boldsymbol{r'}_{m'}) \qquad (17)
$$

where $c_i, c'_j \in \{\mathrm{C}, \mathrm{D}\}$ for $i = 1, 2, \ldots, m$, $j = 1, 2, \ldots, m'$, with m not necessarily equal to m' and $\{\boldsymbol{r}\} \equiv \{\boldsymbol{r}_1, \cdots \boldsymbol{r}_m\}$, $\boldsymbol{c} \equiv \{c_1, \cdots c_m\}$. The primed variables are denoted similarly. Here (m, m') denote the order of the electric field correlation tensor. For brevity, we define the correlation function $M^{m,m'}_\mathrm{out}(\{\boldsymbol{r}\};\{\boldsymbol{r'}\})$ for the output field of an MLI in line with $M^{m,m'}_{\boldsymbol{c};\boldsymbol{c'}}(\{\boldsymbol{r}\};\{\boldsymbol{r'}\})$, except with E_out replacing the field components $E_y^{(c)}$ and $E_y^{(c')}$ in Eq. (17).

The fourth-order correlation $M^{2,2}_\mathrm{out}(\boldsymbol{R};\boldsymbol{R})$ is written explicitly in terms of $M^{2,2}_{\{\mathrm{X,Y}\};\{\mathrm{X',Y'}\}}(\boldsymbol{R};\boldsymbol{R})$ as

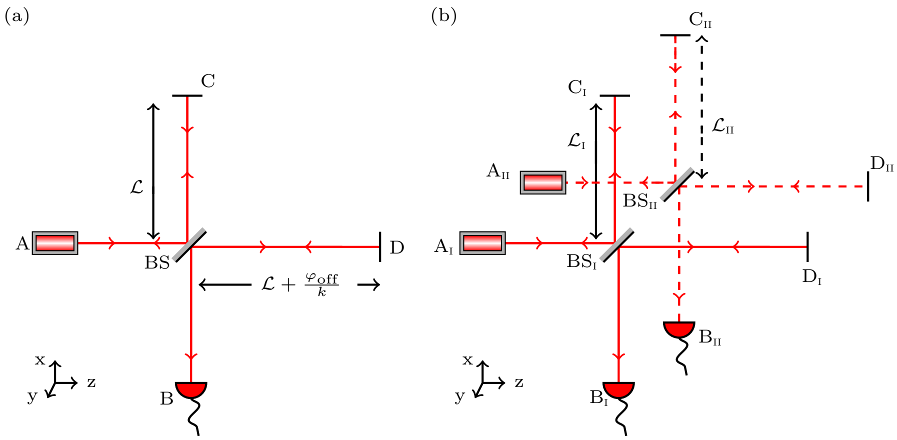

FIG. 1: A schematic diagram of the interferometric setup. (a) Michelson laser interferometer (MLI) with a laser source at the input port A and a detector at the output port B with the two perpendicular arms denoted by C and D. The 50/50 lossless beamsplitter is denoted by BS and is taken as the origin of the reference frame in our computation. We can effectively assume the detector to be at the origin as any change suffered by the light after interference at the BS is common to output field contributions from both the arms and therefore cannot be detected. (b) Two co-located MLIs with input ports A_i and output port B_i with the two perpendicular arms denoted by C_i and D_i each with arm length $\mathcal{L}^i$ ($i=$I,II). We consider $\mathcal{L}^{\mathrm{I}}=\mathcal{L}^{\mathrm{II}}=\mathcal{L}$. The origin is at BS_{I}.

$$
\begin{aligned}
M^{2,2}_{\mathrm{out}}\left(\boldsymbol{R};\boldsymbol{R}\right) = \frac{1}{4}\Bigg[& \sum_{\mathrm{X}\in\{\mathrm{C,D}\}}\sum_{\mathrm{Y}\in\{\mathrm{C,D}\}} M^{2,2}_{\{\mathrm{X,Y}\};\{\mathrm{X,Y}\}}\left(\boldsymbol{R};\boldsymbol{R}\right) - \sum_{\mathrm{X}\in\{\mathrm{C,D}\}} M^{2,2}_{\{\mathrm{X,C}\};\{\mathrm{X,D}\}}\left(\boldsymbol{R};\boldsymbol{R}\right)e^{2i\varphi_{\mathrm{off}}} \\
& - \sum_{\mathrm{X}\in\{\mathrm{C,D}\}} M^{2,2}_{\{\mathrm{X,D}\};\{\mathrm{X,C}\}}\left(\boldsymbol{R};\boldsymbol{R}\right)e^{-2i\varphi_{\mathrm{off}}} - \sum_{\mathrm{X}\in\{\mathrm{C,D}\}} M^{2,2}_{\{\mathrm{C,X}\};\{\mathrm{D,X}\}}\left(\boldsymbol{R};\boldsymbol{R}\right)e^{2i\varphi_{\mathrm{off}}} \\
& - \sum_{\mathrm{X}\in\{\mathrm{C,D}\}} M^{2,2}_{\{\mathrm{D,X}\};\{\mathrm{C,X}\}}\left(\boldsymbol{R};\boldsymbol{R}\right)e^{-2i\varphi_{\mathrm{off}}} + M^{2,2}_{\{\mathrm{C,C}\};\{\mathrm{D,D}\}}\left(\boldsymbol{R};\boldsymbol{R}\right)e^{4i\varphi_{\mathrm{off}}} \\
& + M^{2,2}_{\{\mathrm{D,D}\};\{\mathrm{C,C}\}}\left(\boldsymbol{R};\boldsymbol{R}\right)e^{-4i\varphi_{\mathrm{off}}} + M^{2,2}_{\{\mathrm{D,C}\};\{\mathrm{C,D}\}}\left(\boldsymbol{R};\boldsymbol{R}\right) + M^{2,2}_{\{\mathrm{C,D}\};\{\mathrm{D,C}\}}\left(\boldsymbol{R};\boldsymbol{R}\right)\Bigg]. \qquad (18)
\end{aligned}
$$

We find $\overline{M^{2,2}_{\mathrm{out}}\left(\boldsymbol{R};\boldsymbol{R}\right)}$ with $\boldsymbol{R}=\{(\tau_0,0,0,0),(\tau_0+\Delta_\tau,\Delta,\Delta,\Delta)\}$. We discuss the salient steps involved in computing $\overline{M^{2,2}_{\mathrm{out}}\left(\boldsymbol{R};\boldsymbol{R}\right)}$ by listing the steps in computing one of the terms in this moment, such as,

$$
\overline{M^{2,2}_{\{\mathrm{D,D}\};\{\mathrm{C,C}\}}\left(\boldsymbol{R};\boldsymbol{R}\right)} = |\vec{E}_{in}(0,0)|^2|\vec{E}_{in}(\Delta,\Delta)|^2 \overline{\begin{pmatrix} \exp\left[-i\Omega\left\{\int_0^{\tau_0}\mathrm{d}t'\,w\big(t',0,0,s(t')\big)+\int_{\Delta_\tau}^{\tau_0+\Delta_\tau}\mathrm{d}t'\,w\big(t',\Delta,\Delta,s(t'-\Delta_\tau)+\Delta\big)\right\}\right] \\ \exp\left[i\Omega\left\{\int_0^{\tau_0}\mathrm{d}t'\,w\big(t',s(t'),0,0\big)+\int_{\Delta_\tau}^{\tau_0+\Delta_\tau}\mathrm{d}t'\,w\big(t',\Delta+s(t'-\Delta_\tau),\Delta,\Delta\big)\right\}\right] \end{pmatrix}}. \qquad (19)
$$

Here $s(t)=ct$ if $0\leqslant t\leqslant\tau_0/2$ and $s(t)=2\mathcal{L}-ct$ if $\tau_0/2<t\leqslant\tau_0$. We define this function up to $t\leqslant\tau_0$ when we consider the Holometer. We extend the definition when we consider LIGO.

To simplify $\overline{M^{2,2}_{\mathrm{out}}\left(\boldsymbol{R};\boldsymbol{R}\right)}$, we need to define two correlation integrals for which the following assumptions are required.

(iii) *Stationarity assumption:* $w(\boldsymbol{r})$ is a stationary Gaussian random process with

$$
\overline{w}=0, \quad \text{and} \qquad (20)
$$

$$
\overline{w(t_1,\vec{r}_1)w(t_2,\vec{r}_2)} = \Gamma_{\mathrm{S}}\,\rho\left(ct_{12},\vec{r}_{12}\right). \qquad (21)
$$

Here $o_{12}=o_1-o_2$ ($o=t,\vec{r}$) with $\vec{r}_i\equiv(x_i,y_i,z_i)$ ($i=1,2$) and Γ_{S} is the strength of the fluctuations.

(iv) *Isotropy:* The two-point correlation function ρ is isotropic in space, i.e., $\rho\left(\delta_0,\{\delta_1,\delta_2,\delta_3\}\right)=\rho\left(\delta_0,\{\delta_3,\delta_1,\delta_2\}\right)=\rho\left(\delta_0,\{\delta_3,\delta_2,\delta_1\}\right)=\ldots$, for any separation δ_μ ($\mu=0,1,2,3$). To achieve this isotropy, we consider the correlation to decay with a correlation scale ℓ_r in all three spatial dimensions. We also additionally consider the temporal correlation scale to be ℓ_r/c.

We define the two correlation integrals as follows.

$$\zeta_1(\Delta_\tau,\Delta)=\int\limits_0^{\tau_0}\mathrm{d}t_1\int\limits_0^{\tau_0}\mathrm{d}t_2\,\rho(c(t_1+\Delta_\tau-t_2),\Delta,\Delta,s(t_1)+\Delta-s(t_2))$$

$$=\int\limits_0^{\tau_0}\mathrm{d}t_1\int\limits_0^{\tau_0}\mathrm{d}t_2\,\rho(c(t_1+\Delta_\tau-t_2),s(t_1)+\Delta-s(t_2),\Delta,\Delta), \tag{22a}$$

$$\zeta_2(\Delta_\tau,\Delta)=\int\limits_0^{\tau_0}\mathrm{d}t_1\int\limits_0^{\tau_0}\mathrm{d}t_2\,\rho(c(t_1+\Delta_\tau-t_2),s(t_1)+\Delta,\Delta,\Delta-s(t_2)). \tag{22b}$$

Here the $\sigma(\Delta_\tau)=\zeta_1(\Delta_\tau,0)$ and $\xi(\Delta_\tau)=\zeta_2(\Delta_\tau,0)$.

Using Eqs. (19) and (22), we find

$$\overline{M^{2,2}_{\{\mathrm{C,C}\};\{\mathrm{D,D}\}}}(\boldsymbol{R};\boldsymbol{R})=\overline{M^{2,2}_{\{\mathrm{D,D}\};\{\mathrm{C,C}\}}}(\boldsymbol{R};\boldsymbol{R})=|\vec{E}_{in}(0,0)|^2|\vec{E}_{in}(\Delta,\Delta)|^2\,e^{-\Omega^2\Gamma_\mathrm{S}\{2\zeta_1(0,\Delta)+2\zeta_1(\Delta_\tau,\Delta)-2\zeta_2(0,\Delta)-2\zeta_2(\Delta_\tau,\Delta)\}}$$

$$\approx|\vec{E}_{in}(0,0)|^2|\vec{E}_{in}(\Delta,\Delta)|^2\left[1-\Omega^2\Gamma_\mathrm{S}\left\{2\zeta_1(0,\Delta)+2\zeta_1(\Delta_\tau,\Delta)-2\zeta_2(0,\Delta)-2\zeta_2(\Delta_\tau,\Delta)\right\}\right], \tag{23}$$

$$\overline{M^{2,2}_{\{\mathrm{C,D}\};\{\mathrm{C,D}\}}}(\boldsymbol{R};\boldsymbol{R})=\overline{M^{2,2}_{\{\mathrm{D,C}\};\{\mathrm{D,C}\}}}(\boldsymbol{R};\boldsymbol{R})=|\vec{E}_{in}(0,0)|^2|\vec{E}_{in}(\Delta,\Delta)|^2\,e^{-\Omega^2\Gamma_\mathrm{S}\{2\zeta_1(0,\Delta)-2\zeta_1(\Delta_\tau,\Delta)-2\zeta_2(0,\Delta)+2\zeta_2(\Delta_\tau,\Delta)\}}$$

$$\approx|\vec{E}_{in}(0,0)|^2|\vec{E}_{in}(\Delta,\Delta)|^2\left[1-\Omega^2\Gamma_\mathrm{S}\left\{2\zeta_1(0,\Delta)-2\zeta_1(\Delta_\tau,\Delta)-2\zeta_2(0,\Delta)+2\zeta_2(\Delta_\tau,\Delta)\right\}\right]. \tag{24}$$

$$\overline{M^{2,2}_{\{\mathrm{X,C}\};\{\mathrm{X,D}\}}}(\boldsymbol{R};\boldsymbol{R})=\overline{M^{2,2}_{\{\mathrm{X,D}\};\{\mathrm{X,C}\}}}(\boldsymbol{R};\boldsymbol{R})=\overline{M^{2,2}_{\{\mathrm{C,X}\};\{\mathrm{D,X}\}}}(\boldsymbol{R};\boldsymbol{R})=\overline{M^{2,2}_{\{\mathrm{D,X}\};\{\mathrm{C,X}\}}}(\boldsymbol{R};\boldsymbol{R})$$

$$=|\vec{E}_{in}(0,0)|^2|\vec{E}_{in}(\Delta,\Delta)|^2\,e^{-\Omega^2\Gamma_\mathrm{S}\{\zeta_1(0,\Delta)-\zeta_2(0,\Delta)\}}\approx|\vec{E}_{in}(0,0)|^2|\vec{E}_{in}(\Delta,\Delta)|^2\left[1-\Omega^2\Gamma_\mathrm{S}\left\{\zeta_1(0,\Delta)-\zeta_2(0,\Delta)\right\}\right] \tag{25}$$

The two-point correlation of output power is given by [2]

$$\overline{P^i_\mathrm{out}(\tau)P^j_\mathrm{out}(\tau+\Delta_\tau)}=(\epsilon_0 c)^2\iint\limits_A\mathrm{d}^2a_1\,\mathrm{d}^2a_2\,\overline{M^{2,2}_\mathrm{out}}(\boldsymbol{R};\boldsymbol{R}). \tag{26}$$

Here Eq. (26) can be used for any beam with cross-section area A. However, in our case, we use the assumption that the width of the light beams is effectively zero. In other words, the light beams have been approximated to light rays. Specifically, in Eq. (26), we have used this assumption to simplify the surface integrals to A^2. Further, we also use input power $P_0=2\,\epsilon_0\,c\,A\,|\vec{E}_{in}|^2$ to allow further simplification that leads to Eq. (28) in terms of P_0. Here the covariance of output power is

$$\mathrm{Cov}_{i,j}(P_\mathrm{out})=\overline{P^i_\mathrm{out}(\tau)P^j_\mathrm{out}(\tau+\Delta_\tau)}$$
$$-\overline{P^i_\mathrm{out}(\tau)}\;\;\overline{P^j_\mathrm{out}(\tau+\Delta_\tau)}. \tag{27}$$

Note that $\mathrm{Cov}_{i,j}(P_\mathrm{out})\neq\mathrm{Cov}_{j,i}(P_\mathrm{out})$, in general.

The covariance using Eqs. (25) and (18) for input power P_0,

$$\mathrm{Cov}_{i,j}(P_\mathrm{out})=\frac{P_0^2}{4}\left[\frac{1}{2}\left(1-\Omega^2\Gamma_\mathrm{S}\left\{2\zeta_1(0,\Delta)+2\zeta_1(\Delta_\tau,\Delta)-2\zeta_2(0,\Delta)-2\zeta_2(\Delta_\tau,\Delta)\right\}\right)\cos 4\varphi_\mathrm{off}\right.$$
$$+\frac{1}{2}\left(1-\Omega^2\Gamma_\mathrm{S}\left\{2\zeta_1(0,\Delta)-2\zeta_1(\Delta_\tau,\Delta)-2\zeta_2(0,\Delta)+2\zeta_2(\Delta_\tau,\Delta)\right\}\right)$$
$$\left.-\left(1-\Omega^2\Gamma_\mathrm{S}\left\{2\zeta_1(0,\Delta)-2\zeta_2(0,\Delta)\right\}\right)\cos^2 2\varphi_\mathrm{off}\right]. \tag{28}$$

We know that

$$\mathrm{Cov}_{i,j}(\Delta x) = \left(\frac{\lambda}{4\pi\varphi_{\mathrm{off}}P_0}\right)^2 \mathrm{Cov}_{i,j}(P_{\mathrm{out}}). \tag{29}$$

Using trigonometric identities,

$$\begin{aligned}
\mathrm{Cov}_{i,j}(\Delta x) = \left(\frac{\lambda}{4\pi\varphi_{\mathrm{off}}P_0}\right)^2 \frac{P_0^2}{4}\Bigg[\frac{1}{2}\Bigg(1-\Omega^2\Gamma_{\mathrm{S}}\Big\{2\zeta_1(0,\Delta)+2\zeta_1(\Delta_\tau,\Delta)-2\zeta_2(0,\Delta)-2\zeta_2(\Delta_\tau,\Delta)\Big\}\Bigg)(1-2\sin^2 2\varphi_{\mathrm{off}}) \\
+\frac{1}{2}\Bigg(1-\Omega^2\Gamma_{\mathrm{S}}\Big\{2\zeta_1(0,\Delta)-2\zeta_1(\Delta_\tau,\Delta)-2\zeta_2(0,\Delta)+2\zeta_2(\Delta_\tau,\Delta)\Big\}\Bigg) \\
-\Bigg(1-\Omega^2\Gamma_{\mathrm{S}}\Big\{2\zeta_1(0,\Delta)-2\zeta_2(0,\Delta)\Big\}\Bigg)(1-\sin^2 2\varphi_{\mathrm{off}})\Bigg] \qquad (30) \\
= \left(\frac{\lambda\Omega}{4\pi}\right)^2 \frac{\sin^2 2\varphi_{\mathrm{off}}}{4\varphi_{\mathrm{off}}^2}\Gamma_{\mathrm{S}}\Big[2\zeta_1(\Delta_\tau,\Delta)-2\zeta_2(\Delta_\tau,\Delta)\Big]. \qquad (31)
\end{aligned}$$

Using $\sin^2 2\varphi_{\mathrm{off}} \approx 4\varphi_{\mathrm{off}}^2$ as $\varphi_{\mathrm{off}} \ll 1$,

$$\mathrm{Cov}_{i,j}(\Delta x) = \frac{c^2\Gamma_{\mathrm{S}}}{2}\Big[\zeta_1(\Delta_\tau,\Delta)-\zeta_2(\Delta_\tau,\Delta)\Big]. \tag{32}$$

We need the optical path difference to be stationary to apply the Wiener-Khinchin theorem for obtaining the spectral densities. For each correlation function, we check if the obtained autocorrelation is non-negative definite to check for weak stationarity. We then obtain the PSD using a cosine transform of $\mathrm{Cov}_{i,i}(\Delta x)$ (setting $\Delta = 0$, $\zeta_1 \to \sigma$ and $\zeta_2 \to \xi$) while we obtain the CSD using a Fourier transform of the $\mathrm{Cov}_{\mathrm{I,II}}(\Delta x)$ (setting $\Delta = \Delta_s$).

We simplify the PSD expression as follows. We consider

$$\begin{aligned}
\sigma(\Delta_\tau) = \int_0^{\mathcal{L}/c} \mathrm{d}t_1 \int_0^{\mathcal{L}/c} \mathrm{d}t_2 \Bigg[\rho(c(t_1-t_2+\Delta_\tau), c(t_1-t_2), 0, 0) \\
+\rho\left(c\left(\frac{2\mathcal{L}}{c}-t_1-t_2+\Delta_\tau\right), c(t_1-t_2), 0, 0\right) + \rho\left(c\left(t_1+t_2-\frac{2\mathcal{L}}{c}+\Delta_\tau\right), c(t_1-t_2), 0, 0\right) \\
+\rho(c(t_2-t_1+\Delta_\tau), c(t_1-t_2), 0, 0)\Bigg].
\end{aligned}$$

$$\begin{aligned}
\xi(\Delta_\tau) = \int_0^{\mathcal{L}/c} \mathrm{d}t_1 \int_0^{\mathcal{L}/c} \mathrm{d}t_2 \Bigg[\rho(c(t_1-t_2+\Delta_\tau), ct_1, 0, -ct_2) \\
+\rho\left(c\left(\frac{2\mathcal{L}}{c}-t_1-t_2+\Delta_\tau\right), ct_1, 0, -ct_2\right) + \rho\left(c\left(t_1+t_2-\frac{2\mathcal{L}}{c}+\Delta_\tau\right), ct_1, 0, -ct_2\right) \\
+\rho(c(t_2-t_1+\Delta_\tau), ct_1, 0, -ct_2)\Bigg].
\end{aligned}$$

Applying the cosine transform first over each of the four terms of the two correlation functions and using trignometric identities, we obtain a simplified expression of the PSD. We rewrite this simplified PSD $S(f)$ as $S(\nu)$ in terms of $\nu = \pi f\mathcal{L}/c = \pi f/(2f_{\mathrm{LRT}})$ using $\vec{\Delta}_w = (0, 0, \mathcal{L}(u_1-u_2))$ and $\vec{\Delta}_c = (\mathcal{L}u_1, 0, -\mathcal{L}u_2)$, as

$$S(\nu) = \frac{2\Gamma_{\mathrm{S}}\mathcal{L}^2}{\pi}\int_0^1 \mathrm{d}u_1 \int_0^1 \mathrm{d}u_2 \, \cos(2\nu(1-u_1))\cos(2\nu(1-u_2)) \int_0^\infty \mathrm{d}T \cos\left(2\nu\frac{cT}{\mathcal{L}}\right)\left(\rho(cT,\vec{\Delta}_w)-\rho(cT,\vec{\Delta}_c)\right). \tag{33}$$

III. III. HOLOMETER-TYPE SETUP: RESPONSE FUNCTIONS

In this section, we obtain the interferometer response function for a Holometer-type setup.

We describe an effective phase difference between the two arms of an MLI, given by,

$$\Delta\Phi_{\rm I}\left(\tau_0+\Delta_\tau,\Delta\right)=\varphi_{\rm off}+\Omega\int_{\Delta_\tau}^{\Delta_\tau+\tau_0}{\rm d}t'\left[w(t',s(t'-\Delta_\tau)+\Delta,\Delta,\Delta)-w(t',\Delta,\Delta,s(t'-\Delta_\tau)+\Delta)\right]. \tag{34}$$

We can verify that this effective phase difference gives Eq. (32) multiplied by a factor of $(4\pi/\lambda)^2$, on computing $\mathrm{Cov}_{i,j}(\Delta\Phi_{\rm I}) = \overline{\Delta\Phi_{\rm I}\left(\tau_0,0\right)\Delta\Phi_{\rm I}\left(\tau_0+\Delta_\tau,\Delta\right)} - \overline{\Delta\Phi_{\rm I}\left(\tau_0,0\right)}\ \overline{\Delta\Phi_{\rm I}\left(\tau_0+\Delta_\tau,\Delta\right)}$. Further, by using this effective phase difference, we implicitly assume Attributes (1)-(2) and Assumptions (i)-(iv) listed in Secs. I and II, used in obtaining Eq. (32).

Defining the transform,

$$w(\boldsymbol{r})=w(t,\vec{r}(t))=\int{\rm d}^3\vec{k}_1\int_{-\infty}^{\infty}{\rm d}\omega_1\,\widetilde{w}(\omega_1,\vec{k}_1)\,e^{i\left(\omega_1 t+\vec{k}_1\cdot\vec{r}\right)}, \tag{35}$$

we rewrite Eq. (34) as

$$\Delta\Phi_{\rm I}\left(\tau_0+\Delta_\tau,\Delta\right)=\varphi_{\rm off}+\Omega\int{\rm d}^3\vec{k}_1\int_{-\infty}^{\infty}{\rm d}\omega_1\,\widetilde{w}(\omega_1,\vec{k}_1)\,e^{i\vec{k}_1\cdot\vec{\Delta}_r}\int_{\Delta_\tau}^{\Delta_\tau+\tau_0}{\rm d}t'\left[e^{i\left(\omega_1 t'+\vec{k}_1\cdot\hat{e}_x s(t'-\Delta_\tau)\right)}-e^{i\left(\omega_1 t'+\vec{k}_1\cdot\hat{e}_z s(t'-\Delta_\tau)\right)}\right]. \tag{36}$$

Here $\vec{\Delta}_r=(\Delta,\Delta,\Delta)$. Using the definition of $s(t)$,

$$\int_{\Delta_\tau}^{\Delta_\tau+\tau_0}{\rm d}t' e^{i\left(\omega_1 t'+\vec{k}_1\cdot\hat{e}_x s(t'-\Delta_\tau)\right)}=e^{i\omega_1\Delta_\tau}\left[\left(\frac{e^{i(\omega_1+c\vec{k}_1\cdot\hat{e}_x)\frac{\tau_0}{2}}-1}{i(\omega_1+c\vec{k}_1\cdot\hat{e}_x)}\right)+\left(\frac{e^{\frac{i\omega_1\tau_0}{2}}-e^{i(\omega_1+c\vec{k}_1\cdot\hat{e}_x)\frac{\tau_0}{2}}}{i(\omega_1-c\vec{k}_1\cdot\hat{e}_x)}\right)\right].$$

This implies that

$$\begin{aligned}\Delta\Phi_{\rm I}\left(\tau_0+\Delta_\tau,\Delta\right)=\varphi_{\rm off}+\frac{\Omega\mathcal{L}}{c}\int{\rm d}^3\vec{k}_1\int_{-\infty}^{\infty}{\rm d}\omega_1\,\widetilde{w}(\omega_1,\vec{k}_1)\,e^{i\vec{k}_1\cdot\vec{\Delta}_r}e^{i\omega_1\Delta_\tau}\\ \left[e^{i\frac{\mathcal{L}}{2c}(\omega_1+c\vec{k}_1\cdot\hat{e}_x)}\left\{{\rm Sinc}\left(\frac{\mathcal{L}}{2c}(\omega_1+c\vec{k}_1\cdot\hat{e}_x)\right)+e^{i\omega_1\mathcal{L}/c}{\rm Sinc}\left(\frac{\mathcal{L}}{2c}(\omega_1-c\vec{k}_1\cdot\hat{e}_x)\right)\right\}\right.\\ \left.-e^{i\frac{\mathcal{L}}{2c}(\omega_1+c\vec{k}_1\cdot\hat{e}_z)}\left\{{\rm Sinc}\left(\frac{\mathcal{L}}{2c}(\omega_1+c\vec{k}_1\cdot\hat{e}_z)\right)+e^{i\omega_1\mathcal{L}/c}{\rm Sinc}\left(\frac{\mathcal{L}}{2c}(\omega_1-c\vec{k}_1\cdot\hat{e}_z)\right)\right\}\right]\end{aligned} \tag{37}$$

To obtain $\mathrm{Cov}_{i,j}(\Delta\Phi_{\rm I})$, we find using Eq. (35) that

$$\overline{\widetilde{w}}=0\quad\text{because }\overline{w}=0. \tag{38}$$

$$\overline{w(t_1,\vec{r_1})w(t_2,\vec{r_2})}=\int{\rm d}^3\vec{k}_1\int_{-\infty}^{\infty}{\rm d}\omega_1\int{\rm d}^3\vec{k}_2\int_{-\infty}^{\infty}{\rm d}\omega_2\,\overline{\widetilde{w}(\omega_1,\vec{k}_1)\,\widetilde{w}(\omega_2,\vec{k}_2)}\,e^{i\left(\omega_1 t_1+\vec{k}_1\cdot\vec{r_1}\right)}e^{i\left(\omega_2 t_2+\vec{k}_2\cdot\vec{r_2}\right)}, \tag{39}$$

Here, in Eq. (39), due to stationarity ($\overline{w(t_1,\vec{r_1})w(t_2,\vec{r_2})}$ is only a function of $\vec{r_1}-\vec{r_2}$ and t_1-t_2) we require,

$$\overline{\widetilde{w}(\omega_1,\vec{k}_1)\,\widetilde{w}(\omega_2,\vec{k}_2)}=\Gamma_{\rm s}\widetilde{\rho}(\omega_1,\vec{k}_1)\delta(\omega_1+\omega_2)\delta^{(3)}(\vec{k}_1+\vec{k}_2). \tag{40}$$

Therefore, the covariance of the phase difference becomes

$$\begin{aligned}\mathrm{Cov}_{i,j}(\Delta\Phi_{\rm I})=\Gamma_{\rm s}\left(\frac{\Omega\mathcal{L}}{c}\right)^2\int{\rm d}^3\vec{k}_1\int_{-\infty}^{\infty}{\rm d}\omega_1\,\widetilde{\rho}(\omega_1,\vec{k}_1)\,e^{i\vec{k}_1\cdot\vec{\Delta}_r}\\ \left|e^{i\frac{\mathcal{L}}{2c}(\omega_1+c\vec{k}_1\cdot\hat{e}_x)}\left\{{\rm Sinc}\left(\frac{\mathcal{L}}{2c}(\omega_1+c\vec{k}_1\cdot\hat{e}_x)\right)+e^{i\omega_1\mathcal{L}/c}{\rm Sinc}\left(\frac{\mathcal{L}}{2c}(\omega_1-c\vec{k}_1\cdot\hat{e}_x)\right)\right\}\right.\\ \left.-e^{i\frac{\mathcal{L}}{2c}(\omega_1+c\vec{k}_1\cdot\hat{e}_z)}\left\{{\rm Sinc}\left(\frac{\mathcal{L}}{2c}(\omega_1+c\vec{k}_1\cdot\hat{e}_z)\right)+e^{i\omega_1\mathcal{L}/c}{\rm Sinc}\left(\frac{\mathcal{L}}{2c}(\omega_1-c\vec{k}_1\cdot\hat{e}_z)\right)\right\}\right|^2e^{i\omega_1\Delta_\tau}.\end{aligned} \tag{41}$$

The covariance of the optical path difference is

$$
\begin{aligned}
\mathrm{Cov}_{i,j}(\Delta x) &= \left(\frac{\lambda}{4\pi}\right)^2 \mathrm{Cov}_{i,j}(\Delta\Phi_{\mathrm{I}}) \\
&= \Gamma_{\mathrm{s}}\left(\frac{\mathcal{L}}{2}\right)^2 \int \mathrm{d}^3\vec{k}_1 \int_{-\infty}^{\infty} \mathrm{d}\omega_1\, \widetilde{\rho}(\omega_1,\vec{k}_1)\, e^{i\vec{k}_1\cdot\vec{\Delta}_r} \\
&\left| e^{i\frac{\mathcal{L}}{2c}(\omega_1+c\vec{k}_1\cdot\hat{e}_x)} \left\{ \mathrm{Sinc}\left(\frac{\mathcal{L}}{2c}(\omega_1+c\vec{k}_1\cdot\hat{e}_x)\right) + e^{i\omega_1\mathcal{L}/c}\mathrm{Sinc}\left(\frac{\mathcal{L}}{2c}(\omega_1-c\vec{k}_1\cdot\hat{e}_x)\right)\right\}\right. \\
&\left. -e^{i\frac{\mathcal{L}}{2c}(\omega_1+c\vec{k}_1\cdot\hat{e}_z)} \left\{ \mathrm{Sinc}\left(\frac{\mathcal{L}}{2c}(\omega_1+c\vec{k}_1\cdot\hat{e}_z)\right) + e^{i\omega_1\mathcal{L}/c}\mathrm{Sinc}\left(\frac{\mathcal{L}}{2c}(\omega_1-c\vec{k}_1\cdot\hat{e}_z)\right)\right\}\right|^2 e^{i\omega_1\Delta_\tau}. \qquad (42)
\end{aligned}
$$

The corresponding power spectral density (PSD) with $\Delta=0$ in $\vec{\Delta}_r=(\Delta,\Delta,\Delta)$ is

$$
S(f) = \frac{1}{2\pi}\int_{-\infty}^{\infty} \mathrm{d}\Delta_\tau e^{-2\pi i f\Delta_\tau}\mathrm{Cov}_{i,i}(\Delta x) \qquad (43)
$$

$$
\begin{aligned}
&= \Gamma_{\mathrm{s}}\left(\frac{\mathcal{L}}{2}\right)^2 \int \mathrm{d}^3\vec{k}_1\, \widetilde{\rho}(2\pi f,\vec{k}_1) \left| e^{i\frac{\mathcal{L}}{2c}(2\pi f+c\vec{k}_1\cdot\hat{e}_x)} \left\{ \mathrm{Sinc}\left(\frac{\mathcal{L}}{2c}(2\pi f+c\vec{k}_1\cdot\hat{e}_x)\right) + e^{i2\pi f\mathcal{L}/c}\mathrm{Sinc}\left(\frac{\mathcal{L}}{2c}(2\pi f-c\vec{k}_1\cdot\hat{e}_x)\right)\right\}\right. \\
&\left. -e^{i\frac{\mathcal{L}}{2c}(2\pi f+c\vec{k}_1\cdot\hat{e}_z)} \left\{ \mathrm{Sinc}\left(\frac{\mathcal{L}}{2c}(2\pi f+c\vec{k}_1\cdot\hat{e}_z)\right) + e^{i2\pi f\mathcal{L}/c}\mathrm{Sinc}\left(\frac{\mathcal{L}}{2c}(2\pi f-c\vec{k}_1\cdot\hat{e}_z)\right)\right\}\right|^2. \qquad (44)
\end{aligned}
$$

The correponding cross spectral density (CSD) with a non-zero Δ is

$$
\begin{aligned}
CS(f) &= \frac{1}{2\pi}\int_{-\infty}^{\infty} \mathrm{d}\Delta_\tau e^{-2\pi i f\Delta_\tau}\mathrm{Cov}_{i.j}(\Delta x) \qquad (45) \\
&= \Gamma_{\mathrm{s}}\left(\frac{\mathcal{L}}{2}\right)^2 \int \mathrm{d}^3\vec{k}_1\, \widetilde{\rho}(2\pi f,\vec{k}_1)\, e^{i\vec{k}_1\cdot\vec{\Delta}_r} \\
&\left| e^{i\frac{\mathcal{L}}{2c}(2\pi f+c\vec{k}_1\cdot\hat{e}_x)} \left\{ \mathrm{Sinc}\left(\frac{\mathcal{L}}{2c}(2\pi f+c\vec{k}_1\cdot\hat{e}_x)\right) + e^{i2\pi f\mathcal{L}/c}\mathrm{Sinc}\left(\frac{\mathcal{L}}{2c}(2\pi f-c\vec{k}_1\cdot\hat{e}_x)\right)\right\}\right. \\
&\left. -e^{i\frac{\mathcal{L}}{2c}(2\pi f+c\vec{k}_1\cdot\hat{e}_z)} \left\{ \mathrm{Sinc}\left(\frac{\mathcal{L}}{2c}(2\pi f+c\vec{k}_1\cdot\hat{e}_z)\right) + e^{i2\pi f\mathcal{L}/c}\mathrm{Sinc}\left(\frac{\mathcal{L}}{2c}(2\pi f-c\vec{k}_1\cdot\hat{e}_z)\right)\right\}\right|^2. \qquad (46)
\end{aligned}
$$

We note here that by setting $\Delta=0$ in $\vec{\Delta}_r$, we recover $S(f)$.

Considering that the interferometer response function $\widetilde{\chi}_\Delta(f,\vec{k}_1)$ corresponding to the CSD, is defined using

$$
CS(f) = \int \mathrm{d}^3\vec{k}_1\,\Gamma_{\mathrm{s}}\,\widetilde{\rho}(2\pi f,\vec{k}_1)\,\widetilde{\chi}_\Delta(f,\vec{k}_1), \qquad (47)
$$

we find

$$
\begin{aligned}
\widetilde{\chi}_\Delta(f,\vec{k}_1) = \left(\frac{\mathcal{L}}{2}\right)^2 e^{i\vec{k}_1\cdot\vec{\Delta}_r} &\left| e^{i\frac{\mathcal{L}}{2c}(2\pi f+c\vec{k}_1\cdot\hat{e}_x)} \left\{ \mathrm{Sinc}\left(\frac{\mathcal{L}}{2c}(2\pi f+c\vec{k}_1\cdot\hat{e}_x)\right) + e^{i2\pi f\mathcal{L}/c}\mathrm{Sinc}\left(\frac{\mathcal{L}}{2c}(2\pi f-c\vec{k}_1\cdot\hat{e}_x)\right)\right\}\right. \\
&\left. -e^{i\frac{\mathcal{L}}{2c}(2\pi f+c\vec{k}_1\cdot\hat{e}_z)} \left\{ \mathrm{Sinc}\left(\frac{\mathcal{L}}{2c}(2\pi f+c\vec{k}_1\cdot\hat{e}_z)\right) + e^{i2\pi f\mathcal{L}/c}\mathrm{Sinc}\left(\frac{\mathcal{L}}{2c}(2\pi f-c\vec{k}_1\cdot\hat{e}_z)\right)\right\}\right|^2. \qquad (48)
\end{aligned}
$$

This is rewritten as

$$
\widetilde{\chi}_\Delta(f,\vec{k}_1) = \left(\frac{\mathcal{L}}{2}\right)^2 e^{i\vec{k}_1\cdot\vec{\Delta}_r} \left| C_x(f,\vec{k}_1) - C_z(f,\vec{k}_1)\right|^2, \qquad (49)
$$

with

$$
C_j(f,\vec{k}_1) = e^{ifT_+^{(j)}}\left\{\mathrm{Sinc}\left(fT_+^{(j)}\right) + e^{\frac{2\pi i f\mathcal{L}}{c}}\mathrm{Sinc}\left(fT_-^{(j)}\right)\right\}, \qquad (50)
$$

$$
T_\pm^{(j)}(f,\vec{k}_1) = \frac{\pi\mathcal{L}}{c}\left(1 \pm \frac{c}{2\pi f}\,\vec{k}_1\cdot\hat{e}_j\right), \quad (j=x,z). \qquad (51)
$$

We note that the interferometer response function $\widetilde{\chi}_0(f,\vec{k}_1)$ corresponding to $\Delta = 0$ for obtaining PSD $S(f)$, is denoted simply by $\widetilde{\chi}_{\rm I}(f,\vec{k}_1)$ for ease of notation. Here the PSD is then given by

$$S(f) = \int \mathrm{d}^3\vec{k}_1\, \Gamma_{\rm S}\, \widetilde{\rho}(2\pi f,\vec{k}_1)\, \widetilde{\chi}_{\rm I}(f,\vec{k}_1), \tag{52}$$

with

$$\widetilde{\chi}_{\rm I}(f,\vec{k}_1) = \left(\frac{\mathcal{L}}{2}\right)^2 \left|C_x(f,\vec{k}_1) - C_z(f,\vec{k}_1)\right|^2. \tag{53}$$

IV. IV. LIGO: SPECTRAL DENSITIES AND RESPONSE FUNCTIONS

In this section, we present the signal PSD of the opyical path difference between the two arms of LIGO setup. We also present the interferometer response function in this setup.

LIGO is an MLI with cavities in each arm [3] as shown in Fig. 2. These arm cavities are formed by introducing a mirror in each arm. The electric field at the detector B is

$$E_{\rm out}(\tau,0,0,0) = \tfrac{1}{\sqrt{2}} \sum_{q=1}^{\infty} T_{\rm M}\sqrt{R_{\rm M}^{q-1}} \left[E_y^{(\rm C)}(\tau,0,0,0) - E_y^{(\rm D)}(\tau,0,0,0)e^{-2i\varphi_{\rm off}}\right]. \tag{54}$$

Here $T_{\rm M} = 1 - R_{\rm M}$ is the power transmissivity of the mirrors introduced to render arm cavities. Equation (16) describes the corresponding output electric field in the Holometer-type setup. We use $T_{\rm M} = 0.014$, whereby $R_{\rm M}^{280} \approx 0.019 < 0.02$, i.e., less than 2% of the input light remains after 280 round-trips of the light beam (i.e., finesse of LIGO setup) in each arm.

Using Eq. (54) in place of Eq. (16) and implementing the procedure described in Sec. II (assuming Attributes (1)-(2) and Assumptions (i)-(iv)), we find the PSD to be

$$S(f) = \frac{c^2\Gamma_{\rm S}T_{\rm M}^4}{2}\left(\frac{1}{1-\sqrt{R_{\rm M}}}\right)^2 \sum_{q_1,q_2=1}^{\infty} (\sqrt{R_{\rm M}})^{q_1+q_2-2} \int_0^{\infty} \mathrm{d}\Delta_\tau \left[\sigma^{(q_1,q_2)}(\Delta_\tau) - \xi^{(q_1,q_2)}(\Delta_\tau)\right]\cos 2\pi f\Delta_\tau, \tag{55}$$

where

$$\sigma^{(p,q)}(\Delta_\tau) = \int_0^{p\tau_0}\mathrm{d}t_1\int_0^{q\tau_0}\mathrm{d}t_2\,\rho(c(t_1+\Delta_\tau-t_2),0,0,s(t_1)-s(t_2)) = \int_0^{p\tau_0}\mathrm{d}t_1\int_0^{q\tau_0}\mathrm{d}t_2\,\rho(c(t_1+\Delta_\tau-t_2),s(t_1)-s(t_2),0,0), \tag{56}$$

$$\xi^{(p,q)}(\Delta_\tau) = \int_0^{p\tau_0}\mathrm{d}t_1\int_0^{q\tau_0}\mathrm{d}t_2\,\rho(c(t_1+\Delta_\tau-t_2),s(t_1),0,-s(t_2)). \tag{57}$$

Here $s(t) = ct - 2q\mathcal{L}$ if $q\tau_0 \leqslant t \leqslant \left(\frac{2q+1}{2}\right)\tau_0$ and $s(t) = 2(q+1)\mathcal{L} - ct$ if $\left(\frac{2q+1}{2}\right)\tau_0 < t \leqslant (q+1)\tau_0$.

To obtain the response function in the case of aLIGO, we use the effective phase difference,

$$\Delta\Phi_{\rm L}(\tau_0+\Delta_\tau) = \varphi_{\rm off} + \Omega T_{\rm M}^2\left(\frac{1}{1-\sqrt{R_{\rm M}}}\right)\sum_{q=1}^{\infty}\left(\sqrt{R_{\rm M}}\right)^{q-1} \int_{\Delta_\tau}^{\Delta_\tau+q\tau_0}\mathrm{d}t'\left[w(t',s(t'-\Delta_\tau),0,0) - w(t',0,0,s(t'-\Delta_\tau))\right]. \tag{58}$$

By using this effective phase difference, we implicitly assume Attributes (1)-(2) and Assumptions (i)-(iv) listed in Secs. I and II. We reiterate that these assumptions include all correlation functions that model isotropic, Gaussian spacetime fluctuations (SFs). Implementing the procedure described in Sec. III for the above phase difference we obtain

$$S(f) = \int \mathrm{d}^3\vec{k}_1\, \Gamma_{\rm S}\, \widetilde{\rho}(2\pi f,\vec{k}_1)\, \widetilde{\chi}_{\rm L}(f,\vec{k}_1), \tag{59}$$

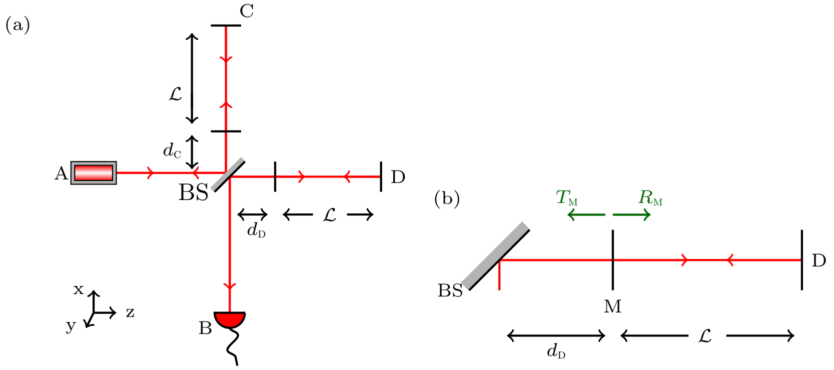

FIG. 2: (a) MLI with arm cavities and $d_{\rm D} - d_{\rm C} = \varphi_{\rm off}/k$, and (b) arm D of the interferometer.

we find

$$\widetilde{\chi}_{\rm L}(f, \vec{k}_1) = \widetilde{\chi}_{\rm I}(f, \vec{k}_1)\, \widetilde{\chi}_{\rm FP}(f), \tag{60}$$

$$\widetilde{\chi}_{\rm FP}(f) = T_{\rm M}^4 \left(\frac{1}{1-\sqrt{R_{\rm M}}}\right)^4 \left(\frac{1}{1+R_{\rm M} - 2\sqrt{R_{\rm M}}\cos(4\pi f \mathcal{L}/c)}\right). \tag{61}$$

Here we recall $\widetilde{\chi}_{\rm I}(f, \vec{k}_1)$ from Eq. (53) and $C_i(f, \vec{k}_1)$ from Eq. (50),

$$\widetilde{\chi}_{\rm I}(f, \vec{k}_1) = \left(\frac{\mathcal{L}}{2}\right)^2 \left|C_x(f, \vec{k}_1) - C_z(f, \vec{k}_1)\right|^2,$$

$$C_j(f, \vec{k}_1) = e^{ifT_+^{(j)}} \left\{\mathrm{Sinc}\left(fT_+^{(j)}\right) + e^{\frac{2\pi i f \mathcal{L}}{c}} \mathrm{Sinc}\left(fT_-^{(j)}\right)\right\},$$

with

$$T_\pm^{(j)}(f, \vec{k}_1) = \frac{\pi \mathcal{L}}{c}\left(1 \pm \frac{c}{2\pi f}\, \vec{k}_1 \cdot \hat{e}_j\right), \qquad (j = x, z).$$

It is evident from Eq. (60) that the response function of LIGO setup factorises into the response functions of the Fabry-Pérot arm cavity and a simple MLI without arm cavities. It is also evident from the above expression that the light-crossing frequency $f_{\rm LRT} = c/(2\mathcal{L})$ is the most dominant frequency scale. We point out that this gain computed, while identical in features to the one obtained in [4, Eq. (A20)], has minor differences due to the following two reasons: (1) we consider interference from two arm cavities instead of a single cavity assumed in Appendix A of [4] and (2) we assume perfect reflectivity of the end mirrors at C and D.

In the limit $\nu \ll 1$, using Taylor series expansion, we find

$$\widetilde{\chi}_{\rm FP}(f) = T_{\rm M}^4 \left(\frac{1}{1-\sqrt{R_{\rm M}}}\right)^6 \left(1 - \frac{16\sqrt{R_{\rm M}}\nu^2}{(1-\sqrt{R_{\rm M}})^2} + \mathrm{O}(\nu^4)\right). \tag{62}$$

Here $\nu = \frac{\pi f}{2 f_{\rm LRT}}$. For $\nu \ll 1$, expanding to $\mathrm{O}(\nu^2)$, we can immediately see that $\widetilde{\chi}_{\rm FP}(f)$ is inversely proportional to ν^2. Further, it is evident that in the limit $\nu \to 0$, the response function of LIGO setup saturates to a product of the gain from the joint effect of the two Fabry-Pérot arm cavities, $T_{\rm M}^4\left(\frac{1}{1-\sqrt{R_{\rm M}}}\right)^6$, and response function of a simple MLI.

V. V. FACTORISED CORRELATION FUNCTION: PSD AND PROPERTIES

In this and the following section, we analyse the trends of the PSD in the case of an MLI at low- and high-frequency limits.

For any 3-vector $\vec{\Delta}_{\rm r}$ and time interval $\Delta_{\rm t}$, the factorised correlation function $\rho_{\rm F}\left(c\Delta_t, \vec{\Delta}_r\right)$ is of the form $\rho_{\rm F}\left(c\Delta_t, \vec{\Delta}_r\right) = \rho_{\rm t}(c\Delta_t)\rho_{\rm s}(\vec{\Delta}_r)$ with ℓ_r setting the correlation scale in space and time. Substituting this into Eq.

(33), it is easily seen that

$$S_{\rm NC}(\nu) = \frac{2}{\pi}\mathcal{S}_{\rm s}(\nu)\mathcal{S}_{\rm t}(\nu), \tag{63}$$

$$\text{with } \mathcal{S}_{\rm s}(\nu) = \int_0^1 {\rm d}u_1 \int_0^1 {\rm d}u_2\, \cos\left(2\nu\left(1-u_1\right)\right)$$
$$\cos\left(2\nu\left(1-u_2\right)\right)\left(\rho_{\rm s}(\vec{\Delta}_{\parallel}) - \rho_{\rm s}(\vec{\Delta}_{\perp})\right), \tag{64}$$

$$\mathcal{S}_{\rm t}(\nu) = \int_0^{\infty} {\rm d}\varphi\, \rho_{\rm t}(\mathcal{L}\varphi)\, \cos 2\nu\varphi. \tag{65}$$

Here, with $t_i = \mathcal{L}u_i/c$ $(i = 1, 2)$, we use $\vec{\Delta}_{\parallel} = (0, 0, s(t_1) - s(t_2))$ and $\vec{\Delta}_{\perp} = (s(t_1), 0, -s(t_2))$. We recall $s(t) = ct$ if $0 \leqslant t \leqslant \tau_0/2$ and $s(t) = 2\mathcal{L} - ct$ if $\tau_0/2 < t \leqslant \tau_0$. Here we note that the temporal shift T (originating from the covariance definition) in Eq. (33) has been scaled suitably to give a dimensionless $\varphi = cT/\mathcal{L}$.

Further, we consider the correlation function $\rho_{\rm s}$ (respectively, $\rho_{\rm t}$) to decay with increase in the spatial (respectively, temporal) separation. We find that the above PSD $S_{\rm NC}(\nu)$ is non-zero at $\nu = 0$ ($S_{\rm NC}(\nu = 0) > 0$) and it decays with increase in scaled freqency ν. This can be concluded using the following arguments. Considering that the *vector magnitude* $\|\vec{\Delta}_{\parallel}\| \leqslant \|\vec{\Delta}_{\perp}\|$ by the geometry of the interferometer with a negligibly small number of points at which the equality is achieved. Therefore, it is evident that $\rho_{\rm s}(\vec{\Delta}_{\parallel}) > \rho_{\rm s}(\vec{\Delta}_{\perp})$ at almost all points. As the other cosine terms in the integral tend to one as $\nu \to 0$, the PSD $S_{\rm NC}(\nu = 0)$ is *non-zero, finite* and positive (PSD needs to be positive by definition). Further, as we expect both ρ_i (i=s,t) to decrease with increase in the corresponding separation, we see that the cosine transforms of such a function will decay with increase in frequency with an appropriate frequency scale.

We can also infer the above from the following mathematical argument. Using the Taylor expansion of the cosine functions in the integrals at $\nu \to 0$, we see that

$$\mathcal{S}_{\rm s}(\nu) = \int_0^1 {\rm d}u_1 \int_0^1 {\rm d}u_2\, \left(\rho_{\rm s}(\vec{\Delta}_{\parallel}) - \rho_{\rm s}(\vec{\Delta}_{\perp})\right)$$
$$\left(1 - 2\nu^2\left((1-u_1)^2 + (1-u_2)^2\right) + {\rm O}(\nu^4)\right), \tag{66}$$

$$\mathcal{S}_{\rm t}(\nu) = \int_0^{\infty} {\rm d}\varphi\, \rho_{\rm t}(\mathcal{L}\varphi)\,(1 - 2\nu^2\varphi^2 + {\rm O}(\nu^4)). \tag{67}$$

We can see that all combinations to order ν^2 shows that the PSD does not increase with increase in frequency at the low-frequency range.

Considering the fact that the factorised correlation function is assumed to decrease with increase in the separation with *finite* correlation scales, we expect the cosine transforms at high frequencies to decay too. So the resulting PSD has an overall decaying trend, barring any local oscillatory behaviour for any general factorised correlation function.

In the specific case of the Oppenheim model, we first note that $S_{\rm NC}(\nu)$ is independent of the choice of $\mathcal{L}$. This is because

$$S_{\rm NC}(\nu) = \frac{2}{\pi}\int_0^1 {\rm d}u_1 \int_0^1 {\rm d}u_2\, \cos\left(2\nu\left(1-u_1\right)\right)$$
$$\cos\left(2\nu\left(1-u_2\right)\right)\left(\sqrt{u_1^2 + u_2^2} - |u_2 - u_1|\right), \tag{68}$$

is evidently independent of $\mathcal{L}$. In the limit $\nu \ll 1$, we find numerically that the logarithmic derivative of the PSD with respect to ν, is $-5\nu/\pi$. For instance, a numerical fit of the PSD in the limit $\nu \ll 1$, yields $S_{\rm NC}(\nu \ll 1) \approx 0.275e^{-\frac{5\nu^2}{2\pi}}$ for $\mathcal{L} = 3$ m. We also find numerically that the scaled PSD $S_{\rm NC}(\nu) \approx \frac{1}{6\nu^2}$ in the limit $\nu \gg 1$.

VI. VI. INVERSE AND EXPONENTIAL CORRELATION FUNCTIONS: PSD AND PROPERTIES

As in the previous section, we examine the PSD at the MLI corresponding to the two classes of correlation functions that cannot be factorised into spatial and temporal parts, at the low- and high-frequency limits.

A. Inverse functions

Using Eq. (33), the scaled PSD corresponding to $\rho_{{\rm I}m}$ ($m = $ S, ST) is

$$S_{\rm C}(\nu) = \frac{2}{\pi}\int_0^1 {\rm d}u_1 \int_0^1 {\rm d}u_2\, \cos\left(2\nu\left(1-u_1\right)\right)$$
$$\cos\left(2\nu\left(1-u_2\right)\right)\left(\mathcal{P}_{{\rm I}m}(\vec{\Delta}_{\parallel}) - \mathcal{P}_{{\rm I}m}(\vec{\Delta}_{\perp})\right). \tag{69}$$

where

$$\mathcal{P}_{\rm IS}(\vec{\Delta}_j) = {\rm sinc}\left(\nu_s^{(j)}\right), \quad \mathcal{P}_{\rm IST}(\vec{\Delta}_j) = \frac{\pi}{2}J_0\left(\nu_s^{(j)}\right), \tag{70}$$

with $\nu_s^{(j)} = 2\nu\|\vec{\Delta}_j\|/\mathcal{L}$ $(j = \parallel, \perp)$ and $J_\alpha(z)$ being the Bessel function of the first kind. It is useful to note that by virtue of the functional forms of $\mathcal{P}_{{\rm I}m}$ and Eq. (69), we find $S_{\rm C}(\nu)$ corresponding to $\rho_{{\rm I}m}$ ($m = $ S,ST) independent of $\mathcal{L}$ and ℓ_r.

Using the Taylor expansion of Eq. (70) about $\nu_s^{(j)} = 0$ $(j = \parallel, \perp)$ in Eq. (69) yields the limiting behaviour of $S_{\rm C}(\nu)$ as $\nu \to 0$.

$$\lim_{\nu\to 0} S_{\rm C}(\nu) = c_{{\rm I}m}\nu^2, \quad (m = {\rm S}, {\rm ST}), \tag{71}$$
$$c_{\rm IS} = 2/(3\pi), \quad c_{\rm IST} = 1/2. \tag{72}$$

Here we note that $S_{\rm C}(\nu = 0) = 0$.

At the high-frequency limit $\nu \gg 1$, it is evident that $\mathcal{P}_{\rm IS}$ (Eq. (70)), which is sinc function, has a $1/\nu$ dependence.

This carries forward to the PSD as a $1/\nu$ dependence in this limit. Similarly, for $\rho_{\rm IST}$, the corresponding $\mathcal{P}_{\rm IST}$ (Eq. (70)) can be approximated in the limit $\nu \gg 1$, as

$$\mathcal{P}_{\rm IST}(\vec{\Delta}_j) \approx \sin\left(\frac{\pi}{4} + 2\nu_s^{(j)}\right) \Big/ 2\sqrt{\pi\nu_s^{(j)}}. \tag{73}$$

It is immediately evident that the spacetime-based $\rho_{\rm IST}$ yields a PSD with $1/\sqrt{\nu}$ dependence in this limit.

B. Exponential functions

The PSD for $\rho_{\rm Em}$ is

$$S_{\rm C}(\nu) = \frac{2\mathcal{L}}{\pi\ell_r}\int_0^1 {\rm d}u_1 \int_0^1 {\rm d}u_2 \, \cos\left(2\nu\left(1-u_1\right)\right) \cos\left(2\nu\left(1-u_2\right)\right)\left(\mathcal{P}_{\rm Em}(\vec{\Delta}_\parallel) - \mathcal{P}_{\rm Em}(\vec{\Delta}_\perp)\right). \tag{74}$$

Here

$$\mathcal{P}_{\rm ES}(\vec{\Delta}_j) = \frac{\|\vec{\Delta}_j\|}{\mathcal{L}}\, e^{-\frac{\mathcal{L}}{\ell_r}\frac{\|\vec{\Delta}_j\|}{\mathcal{L}}}\, {\rm sinc}\left(\nu_s^{(j)}\right), \tag{75}$$

$$\mathcal{P}_{\rm EST}(\vec{\Delta}_j) = \int_0^{\frac{\|\vec{\Delta}_j\|}{\mathcal{L}}} {\rm d}\varphi\, e^{-\frac{\mathcal{L}}{\ell_r}\sqrt{(\|\vec{\Delta}_j\|/\mathcal{L})^2-\varphi^2}} \cos\left(2\,\nu\,\varphi\right), \tag{76}$$

with $\nu_s^{(j)} = 2\nu\|\vec{\Delta}_j\|/\mathcal{L}$ ($j = \parallel, \perp$). As before, we have used the dimensionless $\varphi = cT/\mathcal{L}$ in Eq. (33) to obtain Eq. (76) along with substituting in $\rho_{\rm EST}$. We note that $\vec{\Delta}_j/\mathcal{L}$ depends only on the pair (u_1, u_2), and is independent of $\mathcal{L}$. This clearly implies that Eqs. (75) and (76) depend on the ratio $\kappa = \ell_r/\mathcal{L}$. Therefore, in contrast to the PSDs corresponding to correlation classes considered earlier, $S_{\rm C}(\nu)$ corresponding to $\rho_{\rm Em}$ (m = S,ST) is dependent on this ratio κ.

1. For $\rho_{\rm ES}$

At low frequencies, the Taylor expansion is

$$S_{\rm C}(\nu) = \frac{2}{\pi\ell_r}\int_0^1 {\rm d}u_1 \int_0^1 {\rm d}u_2 \left[\|\vec{\Delta}_\parallel\|\, e^{-\frac{\|\vec{\Delta}_\parallel\|}{\ell_r}} \left(1 - \frac{4\nu^2\|\vec{\Delta}_\parallel\|^2}{3!\mathcal{L}^2}\right) - \|\vec{\Delta}_\perp\|\, e^{-\frac{\|\vec{\Delta}_\perp\|}{\ell_r}}\left(1 - \frac{4\nu^2\|\vec{\Delta}_\perp\|^2}{3!\mathcal{L}^2}\right)\right] \left[1 - \frac{1}{2!}\left(2\nu\left(1-u_1\right)\right)^2 - \frac{1}{2!}\left(2\nu\left(1-u_2\right)\right)^2 + {\rm O}(\nu^4)\right]. \tag{77}$$

It is evident that $S_{\rm C}(\nu = 0)$ is non-zero (and also obviously positive). While it is also evident that this does not increase proportional to ν^2 as in the inverse case, finding an analytical expression for the above integral is not possible. We can, however, resort to numerically finding the logarithmic derivative of the PSD, as before. However, due to the dependence of $S_{\rm C}(\nu)$ on the ratio $\kappa = \ell_r/\mathcal{L}$, the logarithmic derivative also changes with the choice of κ. We find that the logarithmic derivative $S'_{\rm C}(\nu)/S_{\rm C}(\nu) \approx -8\nu/\pi$ for $\kappa = 0.01$. This shows that $S_{\rm C}(\nu \ll 1) \propto e^{-4\nu^2/\pi}$ in this particular case. However, this fit changes significantly for different values of κ.

In the high-frequency limit, the sinc function already indicates a decay with increase in ν. A full analytical expression cannot be obtained. However, we ascertain the high-frequency behaviour as follows. We know that if an expression is a sum of multiple terms with ν^{-q} ($q > 0$) in the limit $\nu \gg 1$, the term most dominant is the one with the smallest q value. So if we identify such a dominant term without solving the full PSD, we could still obtain the behaviour of the PSD at large ν. Using the fact that $\|\vec{\Delta}_\parallel\| \leqslant \|\vec{\Delta}_\perp\|$ by geometry, we know that the terms involving $e^{-\frac{\|\vec{\Delta}_\parallel\|}{\ell_r}}$ would be the dominant terms, when computing the PSD. We consider one such term involved.

$$\frac{1}{\pi\ell_r}\int_0^1 {\rm d}u_1 \int_0^1 {\rm d}u_2 \, \cos\left(2\nu\left(u_2-u_1\right)\right) \|\vec{\Delta}_\parallel\|\, e^{-\frac{\|\vec{\Delta}_\parallel\|}{\ell_r}}\, {\rm sinc}\left(2\nu\|\vec{\Delta}_\parallel\|/\mathcal{L}\right). \tag{78}$$

Using $\|\vec{\Delta}_\parallel\| = \mathcal{L}|u_2 - u_1|$, we find that this simplies to

$$4\frac{\ell_r\mathcal{L}}{\pi}\left(\frac{-2\sqrt{2}\ell_r\mathcal{L}}{\left(\mathcal{L}^2+16\ell_r^2\nu^2\right)^2} + \frac{1}{\left(\mathcal{L}^2+16\ell_r^2\nu^2\right)}\right) + \sqrt{2}\frac{\ell_r\mathcal{L}}{\pi}e^{-\frac{\mathcal{L}}{\sqrt{2}\ell_r}}\left[\frac{8\ell_r\mathcal{L}}{\left(\mathcal{L}^2+16\ell_r^2\nu^2\right)^2}\cos\left(2\sqrt{2}\nu\right) + \frac{\left(\mathcal{L}^2-16\ell_r^2\nu^2\right)}{\nu\left(\mathcal{L}^2+16\ell_r^2\nu^2\right)^2}\sin\left(2\sqrt{2}\nu\right)\right] \tag{79}$$

We can see that the dominant trend in the above term is $\left(\mathcal{L}^2+16\ell_r^2\nu^2\right)^{-1}$. This tallies with the numerical evaluation of the PSD and is illustrated in Fig. 2 (c) in the paper.

2. For $\rho_{\rm EST}$

The PSD expression does not lend itself to analytic simplifications even in the limit cases.

As illustrated in the case of $\rho_{\rm ES}$, here too we expect the behaviour at the low-frequency limit to be an exponential decay. However, obtaining the logarithmic derivative numerically poses challenges and would ultimately be dependent on the ratio κ. So we forego the exercise in this case.

The high-frequency limit is more interesting. Though the higher limit is not analytically tractable, we obtain a

few helpful pointers. It is known that the Fourier cosine transform of $e^{\alpha\sqrt{\varphi^2+\beta^2}}$ ($\mathrm{Re}(\alpha)>0$, $\mathrm{Re}(\beta)>0$) is given in terms of the modified Bessel function of the second kind $K_1(\beta\sqrt{\nu^2+\alpha^2})$ as follows.

$$\int_0^\infty \mathrm{d}\varphi e^{\alpha\sqrt{\varphi^2+\beta^2}}\cos(\varphi\nu) = \alpha\beta(\nu^2+\alpha^2)^{-1/2} K_1(\beta\sqrt{\nu^2+\alpha^2}) \tag{80}$$

Notice the similarity of the LHS of Eq. (80) to the RHS of Eq. (76). Though this does not directly apply here, we expect the limiting behaviour to have some similarity in the gross feature, i.e., for instance, we expect it to tend as $e^{a\nu}/\nu^b$. A numerical fit yields $S_{\mathrm{C}}(\nu)\approx 0.03e^{-0.01}\nu^{-0.3}$ in the limit $\nu\gg 1$. Considering that the arguments used to obtain this fit are not rigorous, we agree that this need not be the correct behaviour. However, we find that the trend is distinctly different from that corresponding to ρ_{ES} or other correlation functions. This $S_{\mathrm{C}}(\nu)$ corresponding to ρ_{EST} is, therefore, still *distinguishable* from those corresponding to other ρ's, even if we cannot identify an analytical limiting behaviour.

VII. VII. RESPONSE FUNCTION APPROACH: OBTAINING $\widetilde{\rho}$

Using Eqs. (39) and (40), we find

$$\widetilde{\rho_{\mathrm{IS}}}(\omega_1,\vec{k}_1) = \frac{\ell_r}{(2\pi)^2\omega_1|\vec{k}_1|}\left[\delta\left(|\vec{k}_1|-\frac{\omega_1}{c}\right)-\delta\left(|\vec{k}_1|+\frac{\omega_1}{c}\right)\right], \tag{81}$$

and

$$\widetilde{\rho_{\mathrm{ES}}}(\omega_1,\vec{k}_1) = \frac{\ell_r^2}{(2\pi)^2\omega_1|\vec{k}_1|}\left[\frac{1-\ell_r^2\left(|\vec{k}_1|-\frac{\omega_1}{c}\right)^2}{4\left(1+\ell_r^2\left(|\vec{k}_1|-\frac{\omega_1}{c}\right)^2\right)^2} - \frac{1-\ell_r^2\left(|\vec{k}_1|+\frac{\omega_1}{c}\right)^2}{4\left(1+\ell_r^2\left(|\vec{k}_1|+\frac{\omega_1}{c}\right)^2\right)^2}\right]. \tag{82}$$

It is evident from Eq. (81) that ρ_{IS} implicitly assumes the wave equation when condsidering the SFs, which becomes apparent in $\widetilde{\rho_{\mathrm{IS}}}$. This also conforms with the Pixellon model where the Pixellon is assumed to satisfy the wave equation [5]. The transformed $\widetilde{\rho_{\mathrm{ES}}}$ is presented for contrast. As we have considered only isotropic models, we also find the transformed correlation functions depend only on the magnitude of $\vec{k}_1$.

VIII. VIII. LIGO: BEHAVIOUR OF PSD

As in Secs. V and VI, we examine the PSD of the output of LIGO, corresponding to two different classes of the correlation function, especially at the low- and high-frequency limits. It is evident from Eq. (60) that the behaviour of the PSD of the output of LIGO can be examined by examining the behaviour of the PSD in the case of the MLI without arm cavities and the Fabry-Pérot cavity response, given by Eq. (61).

Low-frequency limit: In the limit $\nu\ll 1$, the Fabry-Pérot cavity response in Eq. (62) is clearly shown to be inversely proportional to ν^2 and as $\nu\to 0$, this saturates to $T_{\mathrm{M}}^4\left(\frac{1}{1-\sqrt{R_{\mathrm{M}}}}\right)^6$, instead of diverging.

In the case of the correlation function ρ_{IS}, we know from Sec. VI that the PSD of an MLI without arm cavities is directly proportional to ν^2. This implies that when $\nu\ll 1$, the PSD of the output of LIGO is constant with respect to ν. This is because the frequency dependence of the Michelson interferometric response cancels that of the Fabry-Pérot cavity response. However, as $\nu\to 0$, the PSD of LIGO becomes directly proportional to ν^2, because of the saturation in the Fabry-Pérot cavity response.

In the case of ρ_{ES}, it is proportional to an exponential factor which is almost constant in this limit. This implies that the low-frequency behaviour of the Fabry-Pérot cavity response is identical to that of the PSD of LIGO.

High-Frequency limit: In the limit $\nu\gg 1$, the Fabry-Pérot cavity response maximises to $T_{\mathrm{M}}^4\left(\frac{1}{1-\sqrt{R_{\mathrm{M}}}}\right)^6$ at every $4\nu=2m\pi$ ($m\in\{1,2,3,\dots\}$), resulting in peaks at every $f=mf_{\mathrm{LRT}}$. The trend of the MLI response is also carried forward. For instance, this is evident from top panel of Fig. 4 in the paper, where the troughs between the peaks fall as $1/\nu$, which is the behaviour corresponding to the MLI for ρ_{IS}. This is also illustrated in the bottom panel of Fig. 4 in the paper. Here the response remains flat in the range of ν considered due to the significantly smaller r. This behaviour is identical to the PSD correponding to an MLI of the same arm length as LIGO, but with no arm cavities. In both cases, the peaks with the gain $T_{\mathrm{M}}^4\left(\frac{1}{1-\sqrt{R_{\mathrm{M}}}}\right)^6$ render a PSD of the arm strain in LIGO that is significantly larger than the same in table-top interferometers such as QUEST.

SUPPLEMENTARY REFERENCES

[1] C. G. Tsagas, Electromagnetic fields in curved spacetimes, Classical Quant. Grav. **22**, 393 (2004).

[2] B. Sharmila, S. M. Vermeulen, and A. Datta, Extracting electromagnetic signatures of spacetime fluctuations, Classical Quant. Grav. **41**, 075003 (2024).

[3] J. Aasi *et al.* (The LIGO Scientific Collaboration), Advanced LIGO, Classical Quant. Grav. **32**, 074001 (2015).

[4] C. Cahillane and G. Mansell, Review of the Advanced LIGO gravitational wave observatories leading to observing run four, Galaxies **10**, 36 (2022).

[5] D. Li, V. S. H. Lee, Y. Chen, and K. M. Zurek, Interfer-

ometer response to geontropic fluctuations, Phys. Rev. D **107**, 024002 (2023).
